# Impact of biomass fuels on pregnancy outcomes in central East India

**DOI:** 10.1186/1476-069X-13-1

**Published:** 2014-01-09

**Authors:** Blair J Wylie, Brent A Coull, Davidson H Hamer, Mrigendra P Singh, Darby Jack, Kojo Yeboah-Antwi, Lora Sabin, Neeru Singh, William B MacLeod

**Affiliations:** 1 Division of Maternal-Fetal Medicine, Vincent Department of Obstetrics and Gynecology, Massachusetts General Hospital and Harvard Medical School, 55 Fruit Street, Boston, MA, USA; 2 Center for Global Health and Development, Boston University, Boston, MA, USA; 3 Department of International Health, Boston University of School of Public Health, Boston, MA, USA; 4 Department of Environmental Health, Harvard School of Public Health, Boston, MA, USA; 5 Departments of Biostatistics, Harvard School of Public Health, Boston, MA, USA; 6 Department of Medicine, Section of Infectious Diseases, Boston University School of Medicine, Boston, MA, USA; 7 Zambia Centre for Applied Health Research and Development (ZCAHRD), Lusaka, Zambia; 8 National Institute for Malaria Research Field Station, Jabalpur, Madhya Pradesh, India; 9 Department of Environmental Health, Mailman School of Public Health, Columbia University, New York, NY, USA; 10 Regional Medical Research Centre for Tribals (Indian Council for Medical Research), Jabalpur, Madhya Pradesh, India

**Keywords:** Indoor air pollution, Household air pollution, Birth weight, Stillbirth, Preterm birth, Preterm delivery, Pregnancy outcome, Biofuel, Biomass, India

## Abstract

**Background:**

Smoke from biomass burning has been linked to reduced birth weight; association with other birth outcomes is poorly understood. Our objective was to evaluate effects of exposure to biomass smoke on birth weight, preterm birth and stillbirth.

**Methods:**

Information on household cooking fuel was available for secondary analysis from two cohorts of pregnant women enrolled at delivery in India (n = 1744). Birth weight was measured and the modified Ballard performed to assess gestational age. Linear and logistic regression models were used to explore associations between fuel and birth outcomes. Effect sizes were adjusted in multivariate models for socio-demographic characteristics using propensity score techniques and for medical/obstetric covariates.

**Results:**

Compared to women who use gas (n = 265), women cooking with wood (n = 1306) delivered infants that were on average 112 grams lighter (95% CI -170.1, -54.6) and more likely to be preterm (OR 3.11, 95% CI 2.12, 4.59). Stillbirths were also more common in the wood group (4% versus 0%, p < 0.001). In adjusted models, the association between wood use and birth weight was no longer significant (14 g reduction; 95% CI -93, 66); however, the increased odds for preterm birth persisted (aOR 2.29; 95% CI 1.24, 4.21). Wood fuel use did not increase the risk of delivering either a low birth weight or small for gestational age infant.

**Conclusions:**

The association between wood fuel use and reduced birth weight was insignificant in multivariate models using propensity score techniques to account for socio-demographic differences. In contrast, we demonstrated a persistent adverse impact of wood fuel use on preterm delivery. If prematurity is confirmed as a consequence of antenatal exposure to household air pollution, perinatal morbidity and mortality from household air pollution may be higher than previously appreciated.

## Background

Almost half of the world’s population, an estimated 2.8 billion people, relies on solid biomass fuels such as wood, charcoal, crop residues or animal dung for cooking and/or heating [1]. Over a quarter (27%) of the global population using solid fuels reside in India [2]. Smoke released from fires using these fuels emits numerous noxious pollutants, including particulate matter, carbon monoxide, nitrogen dioxide, and carcinogenic organic air pollutants in concentrations that are many-fold higher than those associated with air pollution in polluted cities [3]. Estimates from the recent Global Disease Burden Study 2010 suggest that household air pollution from solid fuel use accounts for 3.5 million premature deaths in children and adults from pneumonia, lung cancer, cardiovascular disease or chronic obstructive pulmonary disease [4]. Household air pollution ranks as the second most important risk factor contributing to disability-adjusted life years lost for women and girls globally, behind only high blood pressure.

In addition to adverse cardiorespiratory effects, there is growing concern about potential perinatal risks associated with biomass burning. An overlap in the constituents of biomass smoke with those of tobacco smoke lends biologic plausibility to a potential adverse impact on pregnancy. Several observational investigations have suggested that maternal exposure to biomass smoke during pregnancy may decrease birth weight and increase the risk for low birth weight (LBW) or stillbirth [5-11]. A systematic review and meta-analysis of these studies (excluding the more recent Epstein publication) reported an increased risk for LBW [summary odds ratio (OR) 1.38, 95% confidence interval (CI) 1.25, 1.52] and stillbirth (summary OR 1.51, 95% CI 1.23, 1.85) as well as a reduction in mean birth weight (-95.6 g, 95% CI -68.5, -124.7) [12]. These effect sizes are similar to those associated with smoking and greater than those attributed to secondhand smoke or ambient air pollution [13-18]. The impact of biomass smoke exposure on preterm delivery is unknown.

We chose to evaluate the relationship between cooking fuel and pregnancy outcome (birth weight, prematurity, and stillbirth) using data from two pregnancy cohorts in India. Information regarding self-reported primary cooking fuel was available for secondary analysis. We hypothesized that women cooking with wood would have smaller babies and an increased risk for stillbirth, LBW, and prematurity.

Women who cook with biomass fuels are likely to be systematically poorer than women who cook with gas. Poverty is linked with adverse pregnancy outcomes through potential mediators such as malnutrition, infections, or poor access to prenatal care. Any identified association between cooking fuel and pregnancy outcomes should therefore consider the possibility of bias from unconsidered variables. We employed propensity score techniques to address this concern and increase power to adjust for multiple potential confounders.

## Methods

### Study sites/procedures

This study is a secondary analysis of data from two cross-sectional cohorts of pregnant women recruited at the time of delivery with the primary aim of defining the burden of malaria in pregnancy in central and east India [19,20]. Case report forms and study procedures were the same in the two cohorts. Data were concatenated to increase power and generalizability. In Jharkhand state, enrollment occurred over a twelve-month period beginning December 2006 at one urban and two rural facilities. In the neighboring state of Chhattisgarh, recruitment occurred from June 2007 to May 2008 in two urban and two rural facilities.

Women aged 15 years or older who presented for delivery at the study sites were enrolled after written informed consent. As part of a background questionnaire focused on socioeconomic and demographic information, the women were interviewed about the primary cooking fuel used in their household and the average number of hours per day they spent cooking during their pregnancy. Additional variables collected during this interview and used for this analysis included information regarding subjects’ socioeconomic status, obstetric histories, medical conditions, as well as pregnancy and delivery complications. Maternal blood pressure, height, and weight were measured upon enrollment prior to delivery. Maternal hemoglobin was determined by capillary finger-stick using a portable HemoCue machine (Ängelholm, Sweden). Peripheral blood was obtained by finger-stick and placental blood by aspiration and impression smear to evaluate for the presence of malaria parasites.

All neonates were weighed with an electronic digital scale to the nearest 10 grams. The gestational ages of all live births were estimated within 24 hours of delivery by means of a modified Ballard examination [21]. Research nurses received both didactic and practical training on the peformance of the Ballard examination by a physician prior to initiation of the study Both neurologic and external features were scored to generate a total Ballard score. The total score was correlated with gestational age using the published Ballard maturity-rating tables (Additional file 1). A preterm delivery was defined as delivery before 37 completed weeks gestation as determined by Ballard examination. Infants were considered small for gestational age (SGA) if their birth weight was less than the 10^th^ percentile for gestational age using an India specific derived reference curve [22], (Additional file 2). Stillbirths were identified by hospital staff as infants without any sign of life at birth. Additional details of the cohorts are presented elsewhere [19,20].

### Data analysis

The distribution of self-reported primary cooking fuels used by enrolled women was determined. The proportions of LBW infants, SGA infants, preterm deliveries, stillbirths, and mean birth weights were compared across the five fuel categories (gas, kerosene, charcoal, wood, and cow dung). Subjects categorizing their primary fuel use as ‘other’ were excluded as were women who delivered more than one infant. Stillbirths were excluded from birth weight analyses (mean birth weight, LBW, SGA). Categorical data are presented as frequency counts (percent) and compared using the Pearson chi-square. Continuous data are summarized as means (± standard deviation) and compared using analysis of variance.

Similar univariate analyses were performed to compare pregnancy outcomes between the two most common fuel types (wood versus gas), used by more than 90% of the sample. It has been recognized that emissions of particulate matter, carbon monoxide and other pollutants may vary by fuel type [3,23,24]; lumping of such fuels into broad categories such as biomass or nonbiomass may be inappropriate. We therefore chose to restrict the subsequent adjusted analyses, outlined below, to a comparison of women cooking with wood to those cooking with gas.

### Propensity score model

To address the systematic differences between those women cooking with wood versus gas, a propensity score model was created [25]. Women who primarily cooked with wood were compared to women cooking with gas across a number of variables that were potentially linked with exposure (biomass cooking fuel) but which might be confounded by poverty. To construct the propensity model, the association of each potential covariate with biomass fuel use was explored; ORs and c-statistics were calculated. The OR, and corresponding 95% CI, represented the odds of using wood given that particular covariate. For example, what are the odds that a subject cooks with biomass fuel if the roof of her house is made of impermanent material? The c-statistic helps identify how well each considered covariate predicts wood fuel use; it incorporates both the strength of the association and the prevalence of the predictor between the wood and gas groups.

Considered variables included *maternal habits*: smoking, tobacco chewing, alcohol use; *adequacy of antenatal care*: 4 or more antenatal visits, use of iron, use of folic acid; and *sociodemographic characteristics*: ownership of common household items, housing characteristics, education, occupation, marital status, and caste. All potential covariates that were significantly associated with wood use in univariate analyses were considered for inclusion in the propensity score model. The variables with the highest c-statistic were included first and an overall c-statistic calculated for the propensity score model. Additional covariates were included in the final model if the c-statistic for the overall model improved. After the final variables for the propensity score model were chosen, a propensity score was calculated for each subject. The propensity score represents the predicated probability that the subject’s primary household fuel was wood (range 0 to 1).

We chose *a priori* to exclude obstetric or medical covariates that were more strongly linked with obstetric outcomes, but considered these variables for the final adjusted models. Covariates excluded from the propensity score model included *maternal characteristics:* age, body mass index, gravidity; *underlying maternal medical conditions:* diabetes, hypertension, HIV status, self-reported anemia; *pregnancy complications:* antenatal vaginal bleeding, self-reported gestational hypertension, self-reported gestational diabetes, fever in week prior to delivery; and *delivery outcomes and complications:* gestational age at delivery, infant gender, measured hypertension at delivery, measured hemoglobin at delivery, and malaria infection. We also excluded time spent cooking and the presence of windows for the propensity model, as these variables might differentiate levels of cook smoke exposure, again leaving these for consideration in the final adjusted models.

### Regression modeling for pregnancy outcomes

The association of fuel type with the categorical outcomes of LBW, SGA, and preterm birth was analyzed using unconditional logistic regression models to estimate univariate ORs and associated 95% CIs. Linear regression models were similarly fit for the outcome of birth weight (as a continuous variable). Exact logistic regression modeling was used to estimate the odds of stillbirth by fuel type given the rarity of the outcome. Gas fuel use was modeled as the referent in all analyses.

Exploratory analyses suggested that the propensity score should be modeled nonlinearly into the outcome models. The propensity score was therefore categorized into quintiles and models subsequently adjusted by the categorized propensity score. Additional sensitivity analyses were performed to determine if models adjusted by the propensity score alone were similar in effect size to a traditional regression model where all covariates from the propensity score were included in the regression. We chose not to match subjects by the propensity score as our cohort was not large enough to sustain the decrement in sample size that would accompany matching.

The exact logistic regression model for stillbirth was adjusted only for propensity score quintiles. All other models were adjusted for cohort effect (Jharkhand versus Chhattisgarh). Additional variables were selected for inclusion in the fully adjusted multivariate models based on their association with the pregnancy outcomes of interest (birth weight, LBW, SGA, preterm birth). If a variable was associated with the specific outcome of interest at a significance level of 0.05 or less, it was included in the final model even if it was not associated with fuel type. For continuous covariates, sensitivity analyses were performed to determine whether a quadratic term was required; these were included in the final adjusted models if inclusion altered the point estimates by 10% or more. We chose not to adjust birth weight models for gestational age as controlling for a potential mediator could eliminate an association. For example, an association between wood fuel use and birth weight may be mediated through early gestational age at delivery. Sensitivity analyses were conducted to determine if inclusion of gestational age in these models altered our conclusions. Statistical analyses were performed using SAS software version 9.2 (Cary, North Carolina).

### Ethical clearance

The study was approved by the Boston University and Centers for Disease Control and Prevention Institutional Review Boards, the Ethics committee and the Scientific Advisory Committee of the National Institute of Malaria Research in India, and the Health Ministry Screening Committee of Indian Council of Medical Research.

## Results

There were 1744 subjects available for secondary analysis from the two pregnancy cohorts. In the state of Jharkhand, recruitment and enrollment into the parent study took place from December 2006 to December 2007. A total of 739 pregnant women were screened at the time of delivery and all were eligible, although 21 refused to provide consent. The 718 remaining had information available on their primary cooking fuel for analysis. In the state of Chhattisgarh, recruitment and enrollment into the parent study took place from June 2007 to May 2008. All 1030 pregnant women screened in DU were eligible. Two women refused to provide consent and another two were missing information regarding their cooking fuel leaving 1026 for this analysis.

The majority of women (1306/1744, 74.9%) used wood in their homes as the primary cooking fuel. The next most common primary fuel reported was gas (265/1744, 15.2%). Charcoal was used by 129 women (7.4%), kerosene by 22 (1.3%), and cow dung by 16 (0.9%). Six women reported their primary fuel as ‘other’.

### Comparison of birth outcomes by primary fuel type

Pregnancy outcomes by the five primary household cooking fuels are summarized in Table 1. There were significant differences between the fuel categories in mean birth weight (p = 0.003), stillbirth (p = 0.01), and preterm delivery (<0.0001). Neither the proportion of LBW infants nor the proportion of SGA infants varied significantly by fuel type (p = 0.09 and p = 0.75 respectively). Examination of Table 1 suggests heterogeneity in pregnancy outcomes by fuel type. The charcoal group appears distinct with fewer infants delivered preterm or stillborn when compared with other biomass fuels. Of note, stillbirths were highest in the kerosene group (9.1%, 2 of 22).

**Table 1:** **Pregnancy outcomes by primary cooking fuel**^**a**^

	**Birth weight (grams)**	**Low birth weight (<2500 grams)**	**Small for gestational age (birth weight <10%)**	**Stillbirth**	**Preterm delivery (<37 weeks)**
Cow dung	2611 ± 403	5/14 (35.7%)	1/14 (7.1%)	1/15 (6.7%)	6/14 (42.9%)
Wood	2623 ± 429	286/1199 (23.9%)	71/1190 (6.0%)	50/1255 (4.0%)	390/1194 (32.7%)
Charcoal	2617 ± 376	36/125 (28.8%)	9/125 (7.2%)	2/127 (1.6%)	15/125 (12.0%)
Kerosene	2716 ± 277	3/20 (15.0%)	1/20 (5.0%)	2/22 (9.1%)	2/20 (10%)
Gas	2736 ± 409	48/253 (19.0%)	20/244 (8.2%)	0/253 (0%)	33/245 (13.5%)

^a^For birth weight outcomes, analyses limited to singleton live births with recorded birth weights. For stillbirths, all singleton births included. For preterm delivery, analyses limited to singleton live births with recorded Ballard examinations. Values represent n(%) or mean ± STD.

### Comparison of wood versus gas users

The distribution of demographic, socioeconomic, obstetric and medical characteristics by fuel type, restricted to wood versus gas, is summarized in Table 2. As anticipated, women using wood differed significantly from those using gas. Women in the wood group weighed significantly less, and were less likely to have attended at least four antenatal visits, less likely to be taking iron and folate, and more likely to chew tobacco during pregnancy or use alcohol compared to gas users. Agricultural work was more common, housing more likely to be constructed of impermanent materials, and years of schooling fewer among women cooking with wood. Ownership of modern amenities was also less frequent in the wood group. At delivery, women primarily using wood were more likely to be hypertensive or anemic. They also were more likely to be members of traditionally disadvantaged populations given administrative recognition: ‘Scheduled Tribes’, originally indigenous people; ‘Scheduled Castes’, primarily consisting of historically lower castes; and ‘Other Backward Castes’, encompassing groups not included in other classifications. Gas users were more likely to be members of the ‘General Caste’, comprised of all other individuals, including higher castes.

**Table 2:** **Distribution of demographic and socioeconomic variables in women cooking with wood versus gas**^**a**^

	**Wood group**	**Gas group**	**Significance**
	**n = 1255**	**n = 253**	
**Maternal characteristics**			
Age^b,^*^,^**			0.0877
< 20 years	104 (8.3%)	13 (5.1%)	
> 20 years	1151 (91.7%)	240 (94.9%)	
BMI at delivery^*,**,#^ (kg/m^2^)	20.6 ± 2.3	21.3 ± 2.3	<0.0001
Gravidity^*,**,#^	2.01 ± 1.3	1.9 ± 1.2	0.1542
**Maternal habits**			
Smoked during pregnancy	2 (0.2%)	0 (0%)	1
Chewed tobacco during pregnancy^*,#^	346 (27.6%)	13 (5.1%)	<0.001
**Socio-demographics**			
Cohort			0.6226
Jharkhand	492 (39.2%)	95 (37.6%)	
Chhattisgarh	763 (60.8%)	158 (62.4%)	
Married	1253 (99.8%)	253 (100.0%)	1.0000
Caste^*,**,#^			<0.0001
Historically disadvantaged^c^	1092 (87.2%)	142 (56.1%)	
General	161 (12.9%)	111 (43.9%)	
Time spent cooking daily^*,**,#^ (hours)	2.85 ± 1.0	2.5 ± 0.9	<0.0001
House has windows^#^	818 (65.2%)	226 (89.3%)	<0.0001
Agricultural work^*,**,#^	294 (23.4%)	7 (2.8%)	<0.0001
Formal schooling ≤ 5 years^*,**,#^	689 (54.9%)	42 (16.6%)	<0.0001
Impermanent/semi-permanent roofing^*,**,#^	1209 (96.3%)	101 (39.9%)	<0.0001
Impermanent/semi-permanent flooring^*,**,#^	1099 (87.6%)	38 (15.0%)	<0.0001
Impermanent/semi-permanent wall material^*,**,#^	1105 (88.0%)	41 (16.2%)	<0.0001
Owns radio^*,**,#^	312 (21.9%)	144 (56.9% )	<0.0001
Owns electric fan^*,**,#^	394 (31.4%)	228 (90.1%)	<0.0001
Owns room cooler^*,**,#^	45 (3.6%)	125 (49.4%)	<0.0001
Owns television^*,#^	400 (31.9%)	400 (88.9%)	<0.0001
Owns refridgerator^*,**,#^	7 (0.6%)	72 (28.5%)	<0.0001
Owns motorcycle^*,**,#^	148 (11.5%)	156 (61.7%)	<0.0001
Owns 4 wheel vehicle^*,**^	14 (1.1%)	29 (11.5%)	<0.0001
**Antenatal care and complications**			
Inadequate antenatal visits (<4)^*,**,#^	826 (66.4%)	93 (36.8%)	<0.0001
Taking iron^*,**,#^	958 (76.4%)	220 (87.0%)	0.0002
Taking folate^*,**,#^	906 (72.3%)	21 (83.0%)	0.0004
Antenatal vaginal bleeding	16 (1.3%)	4 (1.6%)	0.6989
Fever in week prior to delivery	92 (7.4%)	17 (6.8%)	0.7336
**Delivery characteristics and complications**			
Male infant	687 (54.7%)	139 (54.9%)	0.9536
Hypertension at delivery^b,*,#^	256 (20.4%)	66 (26.1%)	0.044
Hemoglobin at delivery^*,**^	10.1 ± 1.8	10.7 ± 1.7	<0.0001
Placental or peripheral parasitemia at delivery^*,**^	45 (3.6%)	6 (2.4%)	0.324
Gestational age at delivery	37.0 ± 1.6	37.7 ± 1.5	<0.0001

^a^Limited to subjects with singleton gestation. Values represent n(%) or mean ± STD.

^b^Age categorized as many women unable to recall their birth date.

^c^Historically disadvantaged castes include Scheduled Caste, Other Backward Caste, and Scheduled Tribes.

^*^Significantly associated with birth weight (95% confidence interval for effect size does not include 0 grams).

^**^Significantly associated with low birth weight (95% confidence interval for odds ratio does not include 1).

^#^Significantly associated with preterm birth (95% confidence interval for odds ratio does not include 1). Gestational age not tested for significance as used to determine whether preterm birth occurred.

In the propensity score analysis, an OR, 95% CI, and c-statistic were calculated for each considered covariate with ‘wood exposure’ (yes versus no) modeled as the outcome. The final propensity model chosen included 17 variables addressing housing, ownership of modern amenities, occupation, habits, education, caste, and prenatal care adequacy. The c-statistic for our final model was 0.952. Each subject was then assigned a propensity score, from 0 to 1 after inputting their individual covariate data. The mean propensity score for women cooking with gas was 0.33 and for women cooking with wood was 0.93. The model statement and distribution of the propensity scores for gas and wood groups are presented in Additional file 3.

In unadjusted analyses, women using wood delivered infants that were on average 112 gram lighter (95% CI -170, -55) and more likely to be preterm (32.7% versus 13.5%; OR 3.11, 95% CI 2.12, 4.59) than gas users (Table 3). Stillbirths were also more frequent among women using wood (4.0% versus 0%; p < 0.001; OR 2.71, 95% CI 0.99, ∞). The odds of delivering a LBW or SGA infant were not significantly higher among women using wood.

**Table 3:** **Pregnancy outcomes comparing women cooking with wood versus gas, unadjusted and adjusted analyses**^**a**^

	**Birth weight**			**Stillbirth**^**b**^	**Preterm delivery (<37 weeks)**
	**Mean birth weight (grams)**	**Low birth weight (<2500 grams)**	**Small for gestational age (birth weight <10%)**		
Gas	2736 ± 409	48/253 (19.0%)	20/244 (8.2%)	0/253 (0%)	33/245 (13.5%)
Wood	2623 ± 429	286/1199 (23.9%)	71/1190 (6.0%)	50/1255 (4.0%)	390/1194 (32.7%)
Effect size (wood versus gas), unadjusted (95% CI)	−112 (-170, -55)	1.33 (0.95, 1.88)	0.71 (0.42, 1.19)	2.71 (0.99, ∞)	3.11 (2.12, 4.59)
Adjusted effect size (95% CI)	−14 (-93, 66)^c^	0.95 (0.58, 1.57)^d^	0.53 (0.23, 1.19)^e^	2.06 (0.08, ∞)^f^	2.29 (1.24, 4.21)^g^

^a^For birth weight outcomes, analyses limited to singleton live births with recorded birth weights. For stillbirths, all singleton births included. For preterm delivery, analyses limited to singleton live births with recorded Ballard examinations. Values represent n(%) or mean ± STD.

^b^ORs and lower confidence interval estimated using exact logistic regression.

^c^Adjusted for propensity score, cohort (Jharkhand versus Chhattisgarh), maternal age, body mass index, squared body mass index, gravidity, hypertension at delivery, hemoglobin at delivery, and time spent cooking.

^d^Adjusted for propensity score, cohort (Jharkhand versus Chhattisgarh), maternal age, body mass index, gravidity, hemoglobin at delivery, and time spent cooking.

^e^Adjusted for propensity score, cohort (Jharkhand versus Chhattisgarh), gravidity, hemoglobin at delivery, fever in week prior to delivery and time spent cooking.

^f^Adjusted for propensity score alone.

^g^Adjusted for propensity score, cohort (Jharkhand versus Chhattisgarh), maternal age, body mass index, gravidity, hypertension at delivery, hemoglobin at delivery, presence of windows, and time spent cooking.

After adjustment for quintiles of propensity score and for medical and obstetric covariates, the reduction in average birth weight for infants born to women using wood was diminished to 14 grams; the difference was no longer significant (95% CI -93, 66). The odds of delivering a LBW infant or SGA infant was not linked with wood fuel use even in the adjusted models. As a sensitivity analysis, gestational age was used to adjust the initial univariate birth weight model; the reduction in mean birth weight from exposure to wood fuel cooking decreased from 112 to 45 grams (95% CI -99, 9) underscoring a potential mediating effect. Therefore, gestational age was not included in the fully adjusted birth weight models (birth weight, LBW, SGA).

In contrast to the birth weight models, the increase in the odds of preterm delivery among women using wood persisted after adjustment. The odds of delivering an infant before 37 weeks was more than two times higher for women cooking with wood (adjusted OR 3.11, 95%CI 2.12, 4.59). Similarly, the point estimate for the odds of stillbirth remained two times higher for mothers cooking with wood after adjustment for the propensity score, although the confidence interval was wide and crossed 1.0.

## Discussion

Our study contributes to the growing literature demonstrating an association between biomass fuel use and adverse pregnancy outcome. To date, whether the reduction in birth weight associated with household air pollution reported in the observational literature reflects an increase in preterm deliveries or a reduction in fetal growth has not been well studied. Our most novel finding was the significant association of wood fuel exposure with preterm delivery, an effect that persisted even after adjustment. In contrast to previously published studies, we had access to estimates of gestational age based on a Ballard examination, and did not rely on maternal recall of menses to date a pregnancy. Reportedly, the Ballard examination has been found to confirm gestational age within a range of two weeks even for premature infants [21,26,27]. Several subsequent studies have suggested that the Ballard may underestimate prematurity [28-30]. Unfortunately, we did not have access to accurate prenatal estimations of gestational age as more than half were unaware of their last menstrual period and ultrasound was not common. If misclassification of preterm delivery occurred in our cohort, it likely did not differ based on the maternal fuel use as the research nurses performing the Ballard examination were unaware of the hypothesis of this secondary analysis. Therefore, any misclassification likely would have biased our results towards the null.

We found a reduction in mean birth weight with wood fuel use that was similar in size to previously published observational studies [6,7,11]. However, after adjustments, the reduction was no longer significant suggesting that use of wood was not independently associated with birth weight after accounting for the numerous socio-demographic and obstetric differences between women using wood and those using gas. This underscores that confounding variables are critical to consider as marked differences among the exposed and unexposed can lead to biased estimates of the exposure effect. The use of the propensity score in our models increased the precision of our effect estimates by allowing adjustment for a number of covariates with a single variable [31]. The propensity score was particularly appealing as our primary focus was the association between wood fuel exposure and pregnancy outcomes, not the association of these other variables with pregnancy outcomes.

In contrast to prior literature, we did not demonstrate an increase in the odds of LBW with wood fuel use in unadjusted or multivariate models [6,10,12]. LBW infants include those that are born prematurely, those born growth-restricted, or both. Consequently, as an outcome measure, LBW has limited utility to identify mechanisms of injury from biomass fuel use. We report LBW for comparison to prior literature but include an analysis of SGA infants as this more accurately captures fetal growth restriction as distinct from preterm birth. In this cohort, SGA infants were not more common among women cooking with wood even in adjusted models. Taken together, our findings suggest that prematurity may be underappreciated as a contributor to reduction in birth weight from household air pollution. There is biologic plausibility to this finding. Air pollution and particulate matter exposure have been linked with inflammation and inflammatory states have been linked with preterm delivery [32,33]. This should be considered exploratory and confirmed in larger observational or interventional studies with improved prenatal gestational age assessment, such as with ultrasound. It is difficult to draw firm conclusions from our data about the risks of fetal death with exposure to biomass fuels given the rarity of stillbirth although suggestive of harm. The variation in stillbirth rates by the five primary fuels, particularly the high prevalence among kerosene users, warrants further investigation.

Strengths of the study design include a digital measurement of birth weight to the nearest 10 grams within 24 hours of delivery, formal assessment of gestational age using the postnatal Ballard examination, and the availability of important socio-demographic, medical and obstetric variables that could confound the fuel exposure-pregnancy outcome association. This included measurement at delivery of a number of birth weight determinants including blood pressure, body mass index, hemoglobin, and parasitemia. Women were unaware of our hypothesis regarding cooking fuel and adverse pregnancy outcome at the time they answered questions about their cooking practices which limits the possibility of recall bias or selection bias. Research staff conducting birth measurements and gestational age assessment were unaware at the time the study was conducted of our interest in household air pollution limiting bias in outcome measurement.

Our most notable limitation was the crude exposure measurement for household air pollution. We used primary cooking fuel to categorize subjects without accounting for the possible use of multiple fuels by a given household. We lacked data on cooking behaviors, with the exception of primary fuel used and time spent cooking. We also had no information on secondhand smoke exposure, which may be common in India [34]. Furthermore, we did not directly measure carbon monoxide, fine particulate matter or other potential pollutants. There is considerable variability in exposure that reflects cooking behaviors, ventilation of cooking areas, season, proximity to high traffic roads, urbanity, and other factors [35]. This variability was not well captured by our dichotomous measurement and may have biased our results towards the null.

Our population was entirely facility-based and the results may not be generalizable to women delivering at home. In the states of both Jharkhand and Chhattisgarh where the study was conducted, over 80 percent of deliveries occur in the home [36,37]. There may be less variability in fuel use among women delivering at home limiting the ability to even evaluate the association of biomass fuels with pregnancy outcome in this group.

Despite a relative lack of causal data between household air pollution and health outcomes, there are large advocacy and policy efforts underway to introduce improved cook stoves to millions of households worldwide based on epidemiologic observations similar to ours [38]. There has been only one published randomized intervention trial measuring the impact of an improved stove on health outcomes [39]. Among the 266 women pregnant at the time of the intervention, a nonsignificant increase of 89 grams in the average birth weight was observed among infants born to women who received a chimney stove [40]. As this study was not designed or powered to assess pregnancy outcomes, many of the pregnant women did not receive the intervention until quite late in gestation limiting the ability to observe an effect on pregnancy outcome. There is thus an urgent need for randomized trials testing the ability of improved stoves to reduce exposure to household air pollutants and improve health outcomes, particularly during pregnancy as women are the primary cooks. Several randomized improved stove trials are underway specifically targeting pregnant women (Nepal Clinicaltrials.gov #NCT00786877, Ghana clinicaltrials.gov #NCT01335490). At least one includes ultrasound determination of gestational age at enrollment (Ghana). In addition to randomized trials, we need repeated measurements of personal exposure to pollutants during pregnancy in order to construct exposure-response curves for adverse pregnancy outcomes. These curves may bolster causal inference and identify thresholds of harm or appropriate targets for exposure reduction.

## Conclusions

This analysis found a significant association between wood fuel use and adverse pregnancy outcome, most notably an increase in the risk of preterm delivery. This association persisted in models that accounted for significant socio-demographic differences between women cooking with wood and those cooking with gas. If prematurity is confirmed as an adverse consequence of antenatal exposure to household air pollution, perinatal morbidity and mortality from household air pollution may be higher than appreciated as preterm infants are particularly vulnerable, especially in resource-limited settings where the majority of biomass fuel use occurs.

## Abbreviations

aOR: Adjusted odds ratio; BW: Birth weight; CI: Confidence interval; GA: Gestational age; LBW: Low birth weight defined as birth weight <2500 grams; OR: Odds ratio; Preterm delivery: Delivery <37 weeks gestational age; Stillbirth: Death of a fetus before delivery; SD: Standard deviation.

## Competing interests

The authors declare that they have no competing interests.

## Authors’ contributions

BJW, BAC, DHH,WBM contributed to the conception and design of the study. BJW, DHH, MPS, NS all participated in study implementation and data collection. BJW, BAC, and WBM performed data analyses and with DHH, DJ, LS, KYA assisted with interpretation of the data. BJW drafted the manuscript. All authors contributed to revisions of the manuscript and read and approved the final manuscript.

## Supplementary Material

Additional file 1 A Standard operating procedure for the new Ballard examination. Click here for file

Additional file 2 Weight percentiles for India. Click here for file

Additional file 3 Final model statement for propensity score. Click here for file

## References

[B1] Pachauri S Brew-Hammond A Barnes DF Bouille DH Gitonga S Modi V Johansson TB, Nakicenovic N, Patwardhan A, Gomez-Echeverri L Energy access for development Global Energy Assessment: Toward a Sustainable Future 2012 New York: Cambridge University Press 1401 1458

[B2] United Nations Development Program-World Health Organization Joint Report The energy access situation in developing sountires: A review focused on least developed countries in sub-Saharan Africa 2009 Nairobi, Kenya: United Nations http://www.who.int/indoorair/publications/energyaccesssituation/en/

[B3] Ezzati M Mbinda BM Kammen DM Comparison of emissions and residential exposure from traditional and improved cookstoves in Kenya Environ Sci Technol 2000 34 4 578 583 10.1021/es9905795

[B4] Lim SS Vos T Flaxman AD Danaei G Shibuya K Adair-Rohani K A comparative risk assessment of burden of disease and injury attributable to 67 risk factors and risk factor clusters in 21 regions, 1990-2010: a systematic analysis for the global burden of disease study 2010 Lancet 2012 380 9859 2224 2260 10.1016/S0140-6736(12)61766-8 23245609 PMC4156511

[B5] Mavalanker DV Gray RH Trivedi CR Risk factors for preterm and term low birthweight in Ahmedabad, India Int J Epdemiol 1992 21 2 263 272 10.1093/ije/21.2.263 1428479

[B6] Boy E Bruce N Delgado H Birth weight and exposure to kitchen wood smoke during pregnancy in rural Guatemala Environ Health Perspect 2002 110 1 109 114 11781172 10.1289/ehp.02110109 PMC1240700

[B7] Mishra V Dai Z Smith K Mika L Maternal exposure to biomass smoke and reduced birth weight in Zimbabwe Ann Epidemiol 2004 14 10 740 747 10.1016/j.annepidem.2004.01.009 15519895

[B8] Mishra V Retherford RD Smith KR Cooking smoke and tobacco smoke as risk factors for stillbirth Int J Environ Health Res 2005 15 6 397 410 10.1080/09603120500288913 16506434

[B9] Siddiquai AR Gold EB Yang X Lee K Brown KH Bhutta ZA Prenatal exposure to wood fuel smoke and low birth weight Environ Health Perspect 2008 116 543 549 10.1289/ehp.10782 18414641 PMC2290983

[B10] Tielsch JM Katz J Thulasirai RD Thulasiraj RD Coles CL Sheeladevi S Exposure to indoor biomass fuel and tobacco smoke and risk of adverse reproductive outcomes, mortality, respiratory morbidity and growth among newborn infants in south India Int J Epidemiol 2009 38 5 1351 1363 10.1093/ije/dyp286 19759098

[B11] Epstein MB Bates MN Arora NK Balakrishnan K Jack DW Smith KR Household fuels, low birth weight, and neonatal death in India: The separate impacts of biomass, kerosene, and coal Int J Hyg Environ Health 2013 216 5 523 532 10.1016/j.ijheh.2012.12.006 23347967

[B12] Pope DP Mishra V Thompson L Siddiqui AR Rehfuess EA Weber M Risk of low birth weight and stillbirth associated with indoor air pollution from solid fuel use in developing countries Epidemiol Rev 2010 32 1 70 81 10.1093/epirev/mxq005 20378629

[B13] Windham GC Eaton A Hopkins B Evidence for an association between environmental tobacco smoke exposure and birthweight: a meta-analysis and new data Perinat Epidemiol 1999 13 1 35 57 10.1046/j.1365-3016.1999.00150.x 9987784

[B14] Maisonet M Brush TJ Correa A Jaakkola JK A review of the literature on the effects of ambient air pollution on fetal growth Environ Res 2004 95 1 106 115 10.1016/j.envres.2004.01.001 15068936

[B15] Srám RJ Binková B Dejmek J Bobak M Ambient air pollution and pregnancy outcome: a review of the literature Environ Health Perspect 2005 113 4 375 382 10.1289/ehp.6362 15811825 PMC1278474

[B16] Stillerman KP Mattison DR Guidice LC Woodruff TJ Environmental exposures and adverse pregnancy outcomes: a review of the science Reprod Sci 2008 15 7 631 650 10.1177/1933719108322436 18836129

[B17] Leonardi-Bee J Smyth A Britton J Coleman T Environmental tobacco smoke and fetal health: systematic review and meta-analysis Arch Dis Child Fetal Neonatal Ed 2008 93 5 F351 F361 10.1136/adc.2007.133553 18218658

[B18] United States Department of Health and Human Services Women and smoking: A report of the Surgeon General 2001 Rockville, Maryland http://www.cdc.gov/tobacco/data_statistics/sgr/2001/complete_report/index.htm

[B19] Hamer DH Singh MP Wylie BJ Yeboah-Antwi K Tuchman J Desai M Burden of malaria in pregnancy in Jharkhand State, India Malaria J 2009 8 210 10.1186/1475-2875-8-210 PMC2744702 19728882

[B20] Singh N Singh MP Wylie BJ Hussain M Kojo YA Shekhar C Malaria prevalence among pregnant women in two districts with differeing endemicity in Chhattisgarh India Malaria J 2012 11 274 10.1186/1475-2875-11-274 PMC3489539 22882903

[B21] Ballard JL Khoury JC Wedig K Wang L Eilers-Walsman BL Lipp R New Ballard score, expanded to include extremely premature infants J Pediatr 1991 119 417 423 10.1016/S0022-3476(05)82056-6 1880657

[B22] Mikolajczyk RT Zhang J Betran AP Souza JP Gülmezoglu AM Merialdi M A global reference for fetal-weight and birthweight percentiles Lancet 2011 377 9780 1855 1861 10.1016/S0140-6736(11)60364-4 21621717

[B23] Bhattacharya SC Albina DO Abdul Salam P Emission factors of wood and charcoal-fired cookstoves Biomass Bioenergy 2002 23 6 453 469 10.1016/S0961-9534(02)00072-7

[B24] Lam NL Smith KR Gauthier A Bates MN Kerosene: a review of household uses and their hazards in low-and middle-income countries J Toxicol Environ Health B Crit Rev 2012 15 6 396 432 10.1080/10937404.2012.710134 22934567 PMC3664014

[B25] Rosenbaum P Rubin DB The central role of the propensity score in observational studies for causal effects Biometrika 1983 70 1 41 55 10.1093/biomet/70.1.41

[B26] Koga Y Fijieda K Matsumoto Y Fujimoto S Hattori T Hagissawa M Tahara Y Nagashima T Mizumoto M Gestational age assessment in Japanese low birthweight infants Acta Paediatr Jpn 1994 36 1 71 74 10.1111/j.1442-200X.1994.tb03133.x 8165913

[B27] Sasidharan K Dutta S Narang A Validity of New Ballard score until 7th day of postnatal life in moderately preterm neonates Arch Dis Child Fetal Neonatal Ed 2009 94 1 F39 F44 19103779 10.1136/adc.2007.122564

[B28] Alexander GR de Caunes F Hulsey TC Tompkins ME Allen M Validity of postnatal assessments of gestational age: a comparison of the method of Ballard et al and early ultrasonography Am J Obstet Gynecol 1992 166 3 891 895 10.1016/0002-9378(92)91357-G 1550159

[B29] Moraes CL Reichenheim ME Validity of neonatal clinical assessment for estimation of gestational age: comparison of new Ballard score with date of last menstrual period and ultrasonography Article in Portuguese Cad Saue Publica 2000 16 1 83 94 10.1590/S0102-311X2000000100009 10738153

[B30] Wylie BJ Kalilani-Phiri L Madanitsa M Membe G Nyirende O Mawindo P Gestational age assessment in malaria pregnancy cohorts: a prospective ultrasound demonstration project in Malawi Malar J 2013 12 183 10.1186/1475-2875-12-183 23734718 PMC3679840

[B31] D’Agostino RB Propensity score methods for bias reduction in the comparison of a treatment to a non-randomized control group Stat Med 1998 17 19 2265 2281 10.1002/(SICI)1097-0258(19981015)17:19<2265::AID-SIM918>3.0.CO;2-B 9802183

[B32] Langrish JP Bosson J Unosson J Muala A Newby DE Mills NL Cardiovascular effects of particulate air pollution exposure: time course and underlying mechanisms J Intern Med 2012 272 3 224 239 10.1111/j.1365-2796.2012.02566.x 22724512

[B33] Vrachnis N Vitoratos N Iliodromiti Z Sifakis S Deligeoroglou E Creatsas G Intrauterine inflammation and preterm delivery Ann NY Acad Sci 2010 1205 118 122 10.1111/j.1749-6632.2010.05684.x 20840262

[B34] Kadir MM McClure EM Goudar SS Garces AL Moore J Onyamboko M Exposure of pregnant women to indoor air pollution: a study from nine low and middle income countries Acta Obstet Gynecol Scand 2010 89 4 540 558 10.3109/00016340903473566 19961275 PMC3928066

[B35] Yadama GN Peipert J Sahu M Biswas P Dyda V Social, economic, and resource predictors of variability in household air pollution from cookstove emissions PLoS One 2012 7 10 e46381 10.1371/journal.pone.0046381 23056293 PMC3463604

[B36] International Institute for Population Sciences (iIPS) District level household and facility survey (DLHS-3), 2007-8 2010 Mumbai, IIPS: India Jharkhand

[B37] International Institute for Population Sciences (IPS) District level household and facility survey (DLHS-3), 2007-8: India 2010 Mumbai, IIPS: Chhattisgarh

[B38] Global Alliance for Clean Cookstoves The Cookstove Storyhttp://www.cleancookstoves.org

[B39] Smith KR McCracken JP Weber MW Hubbard A Jenny A Thompson LM Effect of reduction in household air pollution on childhood pneumonia in Guatemala (RESPIRE): a randomised controlled trial Lancet 2011 378 9804 1717 1726 10.1016/S0140-6736(11)60921-5 22078686

[B40] Thompson LR Bruce N Eskenazi B Diaz A Pope D Smith KR Impact of reduced maternal exposures to wood smoke from an introduced chimney stove on newborn birth weight in rural Guatemala Environ Health Perspect 2011 119 10 1489 1494 10.1289/ehp.1002928 21652290 PMC3230429

